# Machine Learning Analysis to Identify Digital Behavioral Phenotypes for Engagement and Health Outcome Efficacy of an mHealth Intervention for Obesity: Randomized Controlled Trial

**DOI:** 10.2196/27218

**Published:** 2021-06-24

**Authors:** Meelim Kim, Jaeyeong Yang, Woo-Young Ahn, Hyung Jin Choi

**Affiliations:** 1 Department of Biomedical Sciences Seoul National University College of Medicine Seoul Republic of Korea; 2 Department of Psychology Seoul National University Seoul Republic of Korea; 3 Department of Brain and Cognitive Sciences Seoul National University Seoul Republic of Korea; 4 Department of Anatomy and Cell Biology Neuroscience Research Institute Wide River Institute of Immunology Gangwon-do Republic of Korea

**Keywords:** digital phenotype, clinical efficacy, in-app engagement, machine learning analysis, mobile phone

## Abstract

**Background:**

The digital health care community has been urged to enhance engagement and clinical outcomes by analyzing multidimensional digital phenotypes.

**Objective:**

This study aims to use a machine learning approach to investigate the performance of multivariate phenotypes in predicting the engagement rate and health outcomes of digital cognitive behavioral therapy.

**Methods:**

We leveraged both conventional phenotypes assessed by validated psychological questionnaires and multidimensional digital phenotypes within time-series data from a mobile app of 45 participants undergoing digital cognitive behavioral therapy for 8 weeks. We conducted a machine learning analysis to discriminate the important characteristics.

**Results:**

A higher engagement rate was associated with higher weight loss at 8 weeks (*r*=−0.59; *P*<.001) and 24 weeks (*r*=−0.52; *P*=.001). Applying the machine learning approach, lower self-esteem on the conventional phenotype and higher in-app motivational measures on digital phenotypes commonly accounted for both engagement and health outcomes. In addition, 16 types of digital phenotypes (ie, lower intake of high-calorie food and evening snacks and higher interaction frequency with mentors) predicted engagement rates (mean *R^2^* 0.416, SD 0.006). The prediction of short-term weight change (mean *R^2^* 0.382, SD 0.015) was associated with 13 different digital phenotypes (ie, lower intake of high-calorie food and carbohydrate and higher intake of low-calorie food). Finally, 8 measures of digital phenotypes (ie, lower intake of carbohydrate and evening snacks and higher motivation) were associated with a long-term weight change (mean *R^2^* 0.590, SD 0.011).

**Conclusions:**

Our findings successfully demonstrated how multiple psychological constructs, such as emotional, cognitive, behavioral, and motivational phenotypes, elucidate the mechanisms and clinical efficacy of a digital intervention using the machine learning method. Accordingly, our study designed an interpretable digital phenotype model, including multiple aspects of motivation before and during the intervention, predicting both engagement and clinical efficacy. This line of research may shed light on the development of advanced prevention and personalized digital therapeutics.

**Trial Registration:**

ClinicalTrials.gov NCT03465306; https://clinicaltrials.gov/ct2/show/NCT03465306

## Introduction

### Background

The use of mobile tools, such as smartphones, to assist health care systems is rapidly growing in the current era. As the interactions between individuals and digital communities via mobile devices are progressively embedded in human lives, understanding the concept of a *digital phenotype* is also important. A digital phenotype is a collected set of data in a digital system intentionally demonstrated by humans or as a secondary outcome of other activities, influencing human behavior. Specifically, the expanding body of health-related data from mobile devices allows us to address real-world life events with problematic behaviors. For example, data related to the timing and periods of one’s digital footprint can be examined as part of a patient’s features with insomnia [1]. Similarly, data from Google searches can recognize suicidal ideation [2]. To date, digital technologies such as smartphone apps afford moment-by-moment perceptible measurements of a person’s behavior regarding preventive and predictive ways to manage health.

Obtaining app users’ attention is a critical issue related to the app’s potential efficacy for behavior change. The association between intervention exposure and efficacy emphasizes the need for a detailed understanding of user engagement [3]. When we deliver an intervention via a mobile app, the users must actively and frequently engage with mobile apps to succeed within the treatment. Thus, identifying predictive markers that can inform engagement in mobile health (mHealth) interventions could potentially strengthen its effectiveness. Previous studies have found that the involvement of social and gamified components or offering personalized feedback from human factors effectively enhances user engagement for app-based interventions [4,5]. In fact, identifying the major principles that can predict users’ engagement and health outcomes is important for exploring systemic elements to strengthen user engagement in digital intervention. Engagement with digital technology is intricate because it is not stationary but a progressive process [6]. It is also multifaceted in its environment, reflecting the quality of the user’s practice, their communication features, and their willingness to use the app over time or repeatedly [7]. Of special interest to this issue, it is noted that intrinsic motivation is a significant precursor for engagement [8]. Moreover, a wide range of cognitive and emotional states, such as self-interest and self-efficacy, are closely related to the user’s engagement [7]. Therefore, it is important to examine motivation, behavior, emotion, and cognition to understand the changes in users’ engagement and predict clinical outcomes. This will intensify the treatment’s efficacy and find good responders to precision medicine. However, finding the major indicator that predicts who will benefit the most from a digital intervention is insufficient. This resulted in only a minor portion of users obtaining advantages from the digital health care system [9,10]. Thus, it is necessary to explore how comprehensive and multidimensional digital phenotypes detect individual differences and determine user engagement in digital interventions.

Another major issue in the digital era is the interpretation and filtering of data for clinical decisions. Although the rapid growth of digital technologies has led to comprehensive and abundant information about one’s health status, analytical methods to clarify and simplify it have not advanced at a compatible pace [11]. This could be addressed as the main bottleneck in current digital phenotyping studies. Some pioneering research has demonstrated statistical methods to derive insights (which predict outcomes) from various digital phenotypes [12-14]. However, the data are mostly heterogeneous and mixed with structured and unstructured frames containing random sampling, artifacts, and inconsistent completion, making traditional statistical models difficult. This can lead to limited or biased results from the data and a lack of replicability of the conclusions. Compared with conventional analytical methods, machine learning analysis can obtain information from scattered and intricate data, offering insights to promote clinical decision making. A recent study has shown that mortality prediction models using intensive care unit data based on a machine learning approach are superior to conventional methods [15]. Algorithms supporting individual-specific predictions may enhance the usability of machine learning prediction models. This could aid in the adaptation of machine learning models as clinical decision-support tools.

### Objectives

In this study, we aim to investigate multidimensional information at different time points using various assessment methods to monitor and predict the engagement and efficacy of the primary outcome. This study plays a significant role in establishing the most practical and effective mHealth intervention paradigm.

## Methods

### Study Design and Participants

**Figure 1:**
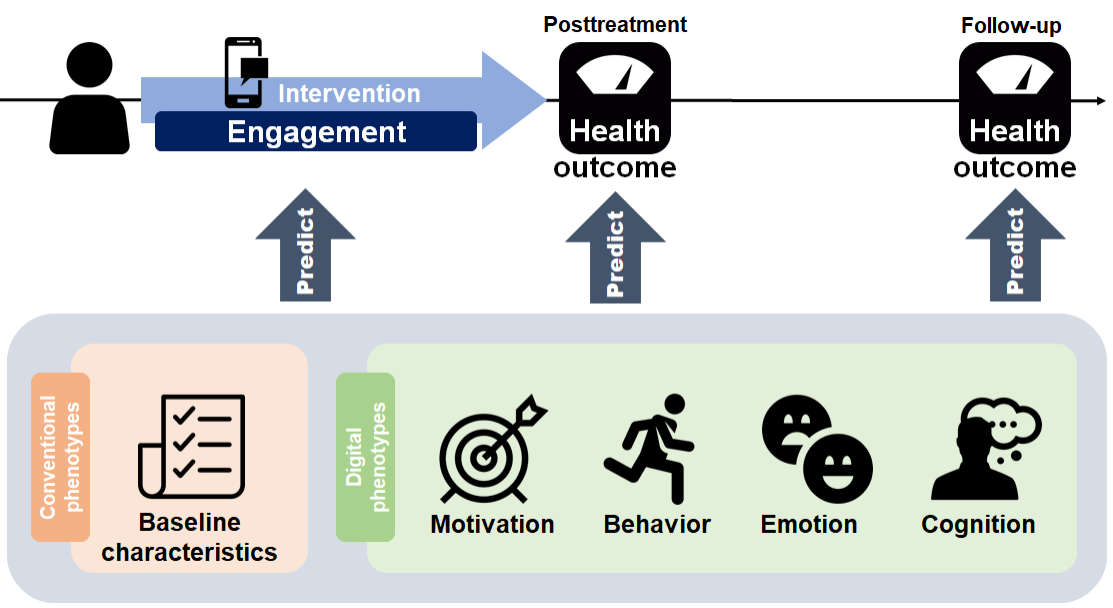
A conceptual framework of mobile health components and examples of digital phenotypes.

We performed a post hoc analysis based on data from a previously reported open-label, 8-week, active comparator randomized controlled trial in the digital cognitive behavioral therapy (dCBT) study. The trial was registered with ClinicalTrials.gov (NCT03465306) in March 2018. Methods of recruitment, inclusion and exclusion criteria, and demographics have been published elsewhere [16]. All study participants provided written informed consent before enrollment in the study. The Institutional Review Board of Seoul National University Hospital approved this study (H-1707-122-872). The study protocol was registered at ClinicalTrials.gov (NCT03465306) on January 15, 2018. This study was conducted to validate the clinical efficacy of the obesity dCBT model and to identify factors related to its efficacy. Furthermore, all the digital phenotypes were averaged for each participant to predict their engagement during the intervention and their health outcomes for both the short term (8 weeks) and long term (24 weeks). A conceptual framework of mHealth components, including examples of digital phenotypes, is presented in Figure 1.

A total of 70 female participants aged between 18 and 39 years, with a BMI of 25-40, eligibility for smartphone use (assessed during the screening interview), and scores in the highest 40% on the Situational Motivation Scale (SIMS; scores above 68 out of 112 in total) were enrolled. We analyzed only 45 participants from the dCBT group. No analysis was performed in the control group. Among the dCBT group, we excluded 6 participants due to dropout, 1 participant due to withdrawal, and 1 participant due to lack of participation (less than 15 days). Therefore, the data analyzed included 37 participants.

### Randomization and Masking

Participants were randomly assigned to a control group or dCBT group with a ratio of 1 to 2 to amplify the power of the dCBT group within analysis and to administer a more robust test within resource restraint. Research participants and research staff were aware of group assignments, but the group assignments were blinded to the technicians and clinical staff.

### Procedures

The aim of this analysis was a post hoc analysis of the effects of a dCBT intervention on obesity. The detailed design and procedures have been described elsewhere [16]. Briefly, the participants in the dCBT group (app+human cognitive behavioral therapy) consisted of daily individualized feedback and assignments from a clinical psychologist based on the CBT modules for 8 weeks. CBT contents were obtained from the program used by the clinicians’ guidelines [17]. The therapist monitored multidimensional components related to the behavior, cognition, emotion, and motivation of each participant in the dCBT group. In contrast, the participants in the control group (app only) were instructed to use the food diary by themselves. All participants were asked to visit at baseline and at 8 and 24 weeks. Anthropometric and self-administered questionnaires were collected at each study visit. The Noom app was mainly used to log food diaries and deliver messages between the therapist and participants.

### Measures

The statistical information for the baseline characteristics and in-app measures is presented in Table 1. There are two main structures: conventional and digital phenotypes, which are classified based on different algorithms. Conventional phenotypes were composed of previously developed and validated surveys. Digital phenotypes are generated by a newly devised scoring system consisting of a combination of active and passive digital features gathered from digital devices. These phenotypes are categorized into four different dimensions: behavioral, cognitive, emotional, and motivational. For conventional phenotypes, four indices for each behavioral and emotional dimension and one index for cognitive and motivational dimensions were assessed. A total of 17 indices for behavioral, 1 for cognitive, 5 for emotional, and 4 for motivational dimensions were assessed regarding digital phenotypes. These categorizations among the four dimensions were proposed based on previous studies [18-21]. The surveys for each dimension were also developed and not validated because they were used only to monitor the users’ condition and not for clinical diagnosis.

**Table 1:** Participant characteristics on demographic, behavioral, cognitive, emotional, and motivational measures.

Phenotype				Value, mean (SD)
**Demographic information**				
	Age (years)			22.59 (3.68)
	Presession BMI			27.86 (3.14)
	Postsession BMI			27.01 (3.51)
**Conventional phenotypes**				
	**Behavioral**			
		Restricted eating (DEBQ-RE^a^)	29.81 (6.90)	
		Emotional eating (DEBQ-EM^b^)	37.54 (9.85)	
		Environmental eating (DEBQ-ENV^c^)	34.76 (4.82)	
		Food addiction (YFAS^d^)	2.54 (1.30)	
	**Cognitive**			
		Automatic thoughts (ATQ-30^e^)	56.92 (21.81)	
	**Emotional**			
		Depression (BDI^f^)	13.22 (8.04)	
		Anxiety (TAI^g^)	47.92 (10.03)	
		Body satisfaction (BSQ-8C^h^)	35.84 (7.30)	
		Self-esteem (RSES^i^)	19.65 (5.15)	
	**Motivational**			
		Conventional motivation (SIMS^j^)	75.97 (5.89)	
**Digital phenotypes**				
	**Behavioral**			
		Carbohydrate	142.95 (26.49)	
		Protein	49.69 (10.60)	
		Fat	38.46 (9.37)	
		Sodium	2190.52 (585.95)	
		Sugar	39.47 (11.42)	
		Breakfast	201.03 (108.33)	
		Morning snack	18.28 (15.98)	
		Lunch	402.16 (98.56)	
		Afternoon snack	56.71 (39.97)	
		Dinner	438.98 (120.26)	
		Evening snack	67.98 (56.61)	
		High-calorie food	0.29 (0.09)	
		Moderate calorie food	0.48 (0.06)	
		Low-calorie food	0.18 (0.09)	
		Steps	6485.00 (2618.54)	
		Exercise	8.17 (8.14)	
		Interaction frequency	9.48 (2.34)	
	**Cognitive**			
		Obesity automatic thoughts	0.49 (0.64)	
	**Emotional**			
		Irritated	46.53 (23.39)	
		Lonely	49.43 (24.52)	
		Nervous	47.26 (23.81)	
		Bored	47.74 (24.18)	
		Depressed	47.04 (24.27)	
	**Motivational**			
		Will	4.58 (2.23)	
		Importance	3.73 (2.10)	
		Confidence	4.11 (2.20)	
		Satisfaction	4.46 (2.40)	

^a^DEBQ-RE: Dutch Eating Behavior Questionnaire–Restricted Eating.

^b^DEBQ-EM: Dutch Eating Behavior Questionnaire–Emotional Eating.

^c^DEBQ-ENV: Dutch Eating Behavior Questionnaire–Environmental Eating.

^d^YFAS: Yale Food Addiction Scale.

^e^ATQ-30: Automatic Thoughts Questionnaire-30.

^f^BDI: Beck Depression Inventory.

^g^TAI: Trait Anxiety Inventory.

^h^BSQ-8C: Body Shape Questionnaire-8C.

^i^RSES: Rosenberg Self-Esteem Scale.

^j^SIMS: Situational Motivational Scale.

Participants’ situational motivation toward the weight loss program was assessed using an adapted version of the SIMS. SIMS typically measures four types of motivation to engage in a task (herein, the weight loss program) at a specific point in time, with four items per subscale: intrinsic motivation, identified regulation, external regulation, and motivation. SIMS has demonstrated acceptable levels of reliability and validity in previous studies. The Body Shape Questionnaire-8C (BSQ-8C) is a brief form of the BSQ and consists of eight items extracted from the full version measuring the extent of the psychopathology of concerns about body shape. Higher BSQ values indicated greater body dissatisfaction. Depression was assessed using the Korean version of the Beck Depression Inventory scoring system. A total score from 0 to 9 indicated no depression, 10 to 15 indicated mild depression, 16 to 23 indicated moderate depression, and 24 to 63 indicated severe depression. Anxiety was measured using the 20-item Trait Anxiety Scale of the State-Trait Anxiety Inventory, with greater scores indicating more trait anxiety. The Rosenberg Self-Esteem Scale measure of self-esteem was used in this research with a 10-item scale with all negatively worded items. Thus, higher scores implied lower self-esteem. Eating behavior was measured using the Dutch Eating Behavior Questionnaire, which has three different psychologically based eating behaviors: restrained eating, emotional eating, and external eating. It contains 33 items, with higher scores indicating a greater tendency to present subscale behavior. The frequency of automatic negative thoughts associated with depression was assessed using the Automatic Thoughts Questionnaire-30. The scores ranged from 30 to 150, and higher scores implied that the participants experienced automatic negative thoughts more often. All psychological questionnaires were presented in Korean.

Six types of behavioral phenotypes, modified and extended from previous studies [22,23], were assessed in apps: food restriction, overeating and binge eating, late-night meals, snacking, food choice, and activity rate. Food restriction was evaluated using calories per meal per day. Overeating and binge eating were assessed by calories per meal per day and the speed per meal—the late-night meal was investigated using the dinner calories and the time per meal. Snacking was estimated using snack calories. Food choice was examined based on the type of food per meal, total amount of sodium and sugar, number of food types per meal, and percentage of nutritional types (carbohydrate, protein, and fat). The activity rate was measured as the number of steps and the total hours of exercise. Automatic thoughts were grouped into six categories: selective abstraction, arbitrary inference, overgeneralization, magnification or minimization, personalization, and absolutism. There were 20 automatic thoughts, and participants could add thoughts related to food or eating behaviors. Example statements for automatic thoughts are listed in Table S1 in Multimedia Appendix 1. We assessed 5 negative emotions closely related to problematic eating habits: irritation, loneliness, nervousness, boredom, and depression. The participants were asked to report each type of negative emotion score using a visual analog scale between 0 and 100. Motivation was assessed using four dimensions: will, rank of importance, confidence, and satisfaction. These different types of motivation were scored using a 10-point Likert scale (1-10).

### Outcomes

The primary outcomes were changes in body weight and number logged into the app. Body weight was assessed by InBody H20B (InBody Co, Ltd) at baseline and 8 and 24 weeks in light street clothing and without socks and shoes. The number logged into the app was examined by tracking the actions such as responses to the daily assessment (responses per day), meals logged (meals per week), green foods defined by Noom (logged per week), exercise logged (times per week), exercise time registered (minutes per week), steps recorded (steps per week), weigh-ins logged (times per week), articles read (articles per week), group posts (posts per week), group comments (comments per week), messages sent to coaches (messages per week), and group likes (likes per week). The engagement rate was assessed using these objective indices for each participant.

### Statistical Analysis

We analyzed the data to predict three target outcomes: (1) the number of mobile activities during the experiment session, (2) the weight change rate between presession (week 0) and postsession (week 8), and (3) the weight change rate between postsession and follow-up. The weight change rates were calculated as the ratio of the weight difference to the baseline weight as (weight_before_–weight_after_)/weight_before_. Correlations between the number of logs and weight change rates were analyzed to determine the relationship between engagement and health outcomes.

A machine learning approach using an elastic net was conducted [24]. The elastic net is a penalized regression method that automatically selects significant variables by reducing the regression coefficients of unimportant features to zero. Using 37 behavioral, cognitive, motivational, and emotional measures, we tried to reveal which measure contributes to predicting behavioral changes before and after treatment.

The analysis procedure for the out-of-sample regressions was similar to that in a previous study [25,26]. To conduct out-of-sample regression, we used leave-one-out cross-validation, which trains a model with data except for a single point and then evaluates the point’s prediction. The root mean squared errors (RMSE) computed for all possible train test splits are averaged to the leave-one-out cross-validation error, which is a measure for evaluating the model fit.

To acquire generalizable coefficients, we conducted model fitting 1000 times for each possible α value, which is the ratio between the ridge and lasso penalty terms. The number of iterations was chosen according to previous literature using a similar approach [25,26]. Figure S1 in Multimedia Appendix 1 shows the RMSE with 100 α values (from 0.01 to 1 with an interval of 0.01), and we chose the α value that minimizes RMSE across all participants. Then, to identify predictors for engagement and health outcomes, we computed mean β coefficients across 1000 iterations, and only phenotypes that were significant in more than 5% of 1000 iterations were selected as predictors for each model [25,26].

## Results

### Relationship Between the Number of Logs and Weight Changes

**Figure 2:**
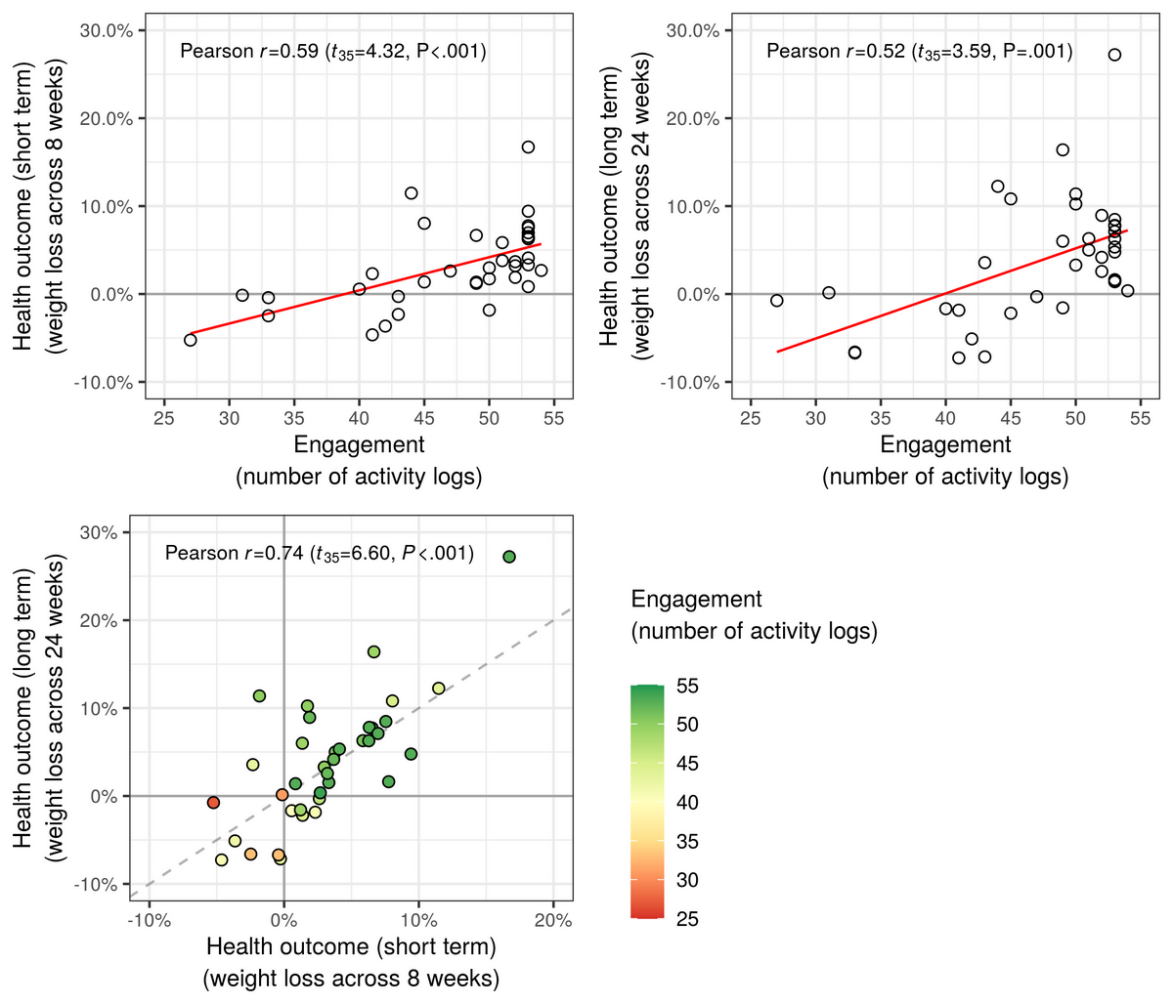
Relationships between engagement and health outcomes. The health outcome larger than zero indicates weight loss compared with baseline.

Figure 2 shows the correlations between the number of logs (engagement) and weight change (health outcomes). For the weight change during the 8-week intervention, two variables were highly correlated (*r*=−0.59; two-tailed t_35_=−4.32; *P*<.001; Figure 2), which indicates that participants who had engaged in the in-app activity more actively lost weight. This result was the same for the weight change between baseline and follow-up (*r*=−0.52; two-tailed t_35_=−3.59; *P*<.001). These short-term and long-term health outcomes were highly correlated (*r*=0.74; two-tailed t_35_=6.60; *P*<.001).

In addition, we tried to check whether there exist nonlinear relationships between the log number of activity logs and short-and long-term health outcomes. Such log transformations did not show significant differences for the relationship with short-term health outcomes (*r_log_*=0.58; z_34_=0.7843; *P*=.43) and even for those with long-term health outcomes (*r_log_*=0.50; z_34_=1.4579; *P*=.14).

### Elastic Net Results

Through the leave-one-out cross-validations with different values for the mixing parameter (α), we chose the best value for each model that showed the minimum RMSE between the data and predicted outcomes. The estimated mixing parameters, α, were .08, .15, and .53 for predicting engagement, short-term health outcome, and long-term health outcome, respectively (Figure S1 in Multimedia Appendix 1). The α estimate for the long-term health outcome was much higher than that in the other two models, suggesting that the multivariate pattern is more parsimonious. Its coefficients are prone to shrink to zero while predicting long-term weight changes.

**Figure 3:**
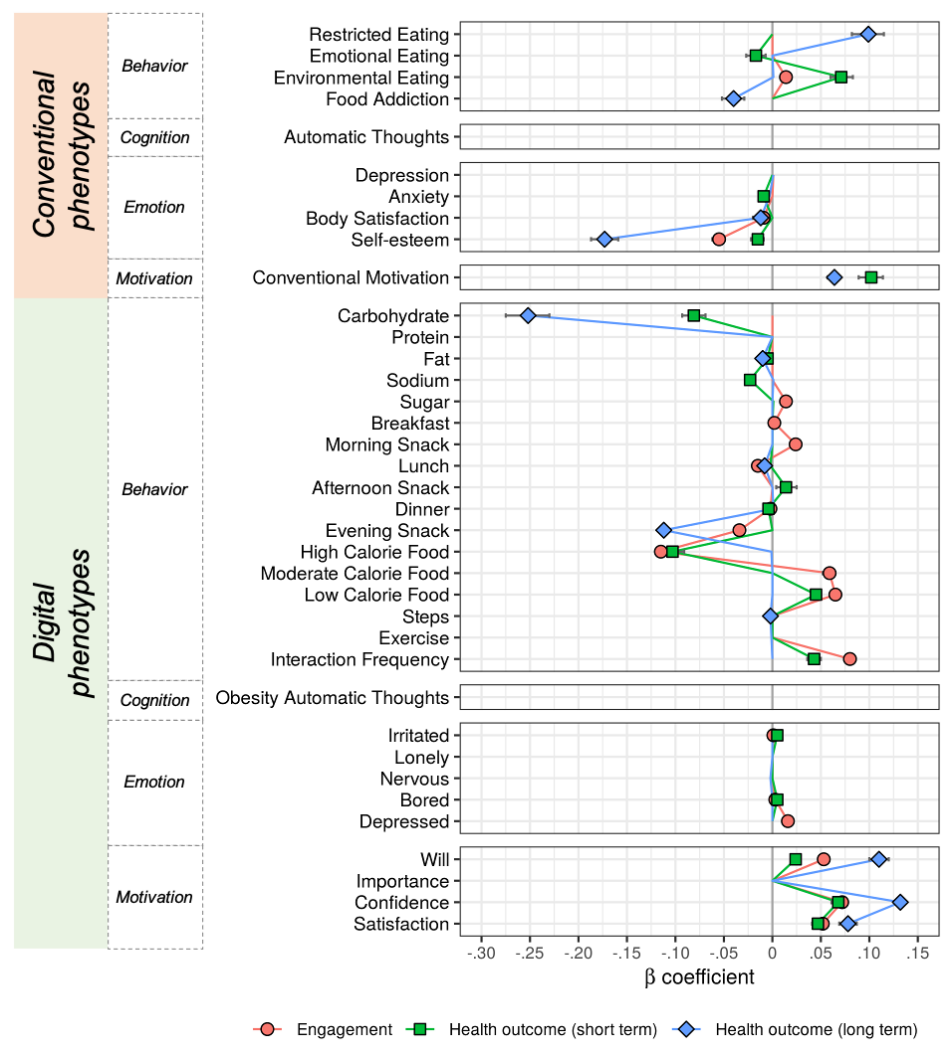
Multivariate patterns of conventional and digital phenotypes for predicting engagement (red) as well as short-term (green) and long-term (blue) health outcomes. Points indicate the averaged β coefficients across 100 repetitions of net elastic analysis (see the Methods section for details). A positive β estimate of a phenotype indicates an association between the phenotype and higher in-app activities (engagement) or more weight loss (health outcomes). The points, which contain zero in the simulated 95% ranges, are omitted.

Figure 3 illustrates the multivariate profiles of conventional and digital phenotypes to predict in-app engagement and the health outcomes of digital health care. In-app engagement, computed as the number of daily activity logs, was significantly associated with lower self-esteem, lower body satisfaction, and higher external eating behaviors, measured as conventional phenotypes. For digital phenotypes, engagement was predicted by lower intake of food with a high calorie density index (CDI), higher food intake in the morning (breakfast and morning snack), lower food intake after that (lunch, dinner, and evening snack), higher sugar intake, higher intake of moderate or low CDI food, and higher frequency of interactions with the therapist. Higher emotional and motivational measures in digital phenotypes were also involved, such as irritation, boredom, depression, satisfaction, will, and confidence.

For short-term health outcomes, lower emotional eating behavior, lower self-esteem, lower anxiety, higher external eating behavior, and higher motivation predicted the weight change rate for 8 weeks. The 8-week weight change was also predicted by lower intake of high CDI food, lower carbohydrate, lower sodium, lower fat intake, higher afternoon snack intake, lower dinner intake, higher intake of low CDI food, and higher frequency interactions with a health care mentor. Furthermore, short-term health outcomes were positively associated with emotional and motivational features in digital phenotypes, such as boredom, irritation, will, satisfaction, and confidence.

In contrast, fewer phenotypes are involved in the prediction of long-term health outcomes. Lower self-esteem, lower food addiction, lower body satisfaction, higher motivation, and higher restricting eating behavior in conventional phenotypes predicted the 24-week weight change. For digital phenotypes, the long-term health outcome was predicted by lower carbohydrate intake, lower lunch and evening snack intake, lower fat intake, lower steps in a day, higher satisfaction, higher will, and higher confidence.

**Figure 4:**
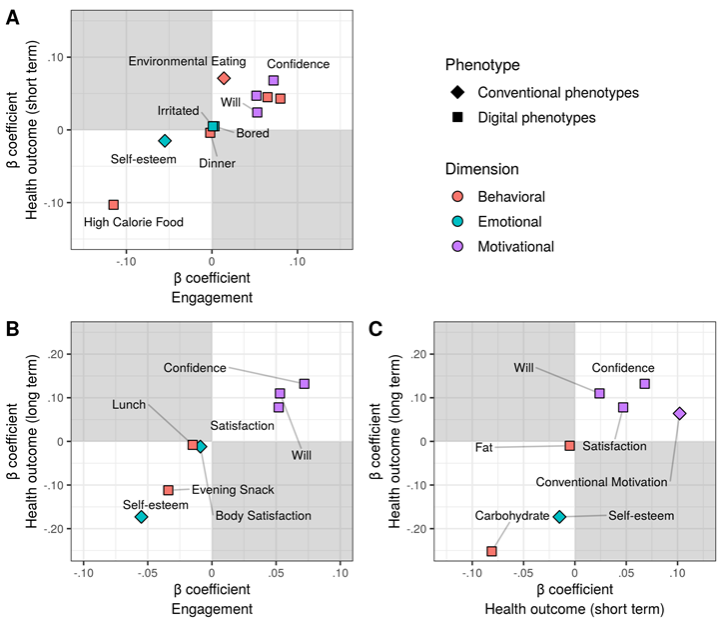
Common predictors between engagement and health outcomes for (A) health outcome (short term) versus engagement, (B) health outcome (long term) versus engagement, and (C) health outcome (long term) versus health outcome (short term). Each axis indicates the β estimate for predicting engagement and health outcomes. A positive β coefficient indicates a positive association with engagement but negative associations with health outcomes (weight changes).

**Figure 5:**
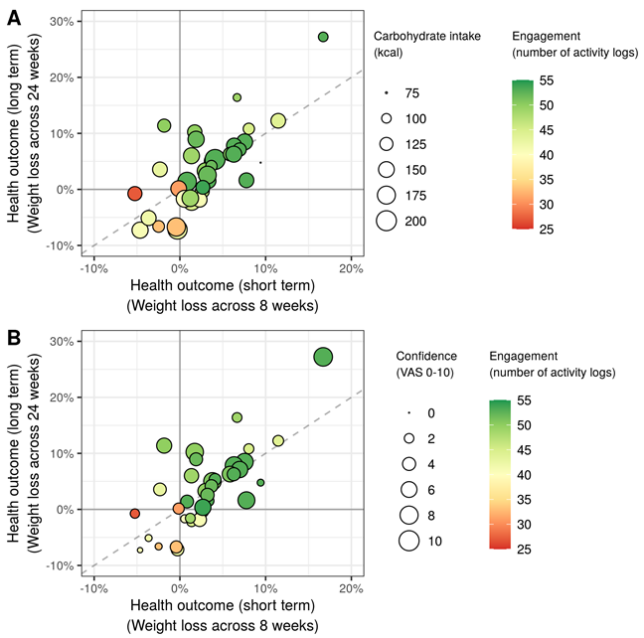
Two examples of common predictors between short-term and long-term health outcomes: (A) carbohydrate intake and (B) confidence in digital phenotypes. VAS: Visual Analogue Scale.

Common predictors across dependent variables were associated with different phenotypes (Figure 4 and Table 2). Engagement and health outcomes were commonly affected by lower self-esteem in conventional phenotypes and higher in-app motivational measures in digital phenotypes. In other words, decreased self-esteem before the intervention and inclined motivation during the intervention highly predicted more in-app activities and more weight loss following the intervention. Furthermore, common predictors between engagement and short-term health outcomes include the behavioral dimension of digital phenotypes, such as the frequency of coach interaction and low- or high-calorie food intake. Carbohydrate intake was the most commonly influential predictor of short-term and long-term health outcomes. Conversely, conventional and digital phenotypes’ motivational measures were positively associated with health outcomes (Figure 5).

**Table 2:** Common and specific predictors of conventional and digital phenotypes for predicting engagement and health outcomes.

Phenotypes			Common predictors^a^		Predictors specific to each dependent variable				
					Engagement		Health outcome (short term)		Health outcome (long term)
**Conventional phenotypes**									
		Self-esteem^b^			Body satisfaction^b^ Environmental eating^c^		Emotional eating^b^ Anxiety^b^ Environmental eating^c^ Conventional motivation^c^		Food addiction^b^ Body satisfaction^b^ Conventional motivation^c^ Restrictive eating^c^
**Digital phenotypes**									
	**Behavioral**								
		N/A^d^		High-calorie food^b^ Night snack^b^ Lunch^b^ Dinner^b^ Breakfast^c^ Sugar^c^ Morning snack^c^ Moderate calorie food^c^ Low-calorie food^c^ Interaction frequency^c^		High-calorie food^b^ Carbohydrate^b^ Sodium^b^ Fat^b^ Afternoon snack^b^ Low-calorie food^c^ Interaction frequency^c^		Carbohydrate^b^ Night snack^b^ Lunch^b^ Fat^b^ Steps^b^	
	**Emotional**								
		N/A		Irritated^c^ Bored^c^ Depressed^c^		Irritated^c^ Bored^c^		N/A	
	**Motivational**								
		Satisfaction^c^ Will^c^ Confidence^c^		N/A		N/A		N/A	

^a^Common predictors in the first column were involved in all models. The cognitive dimension of digital phenotypes was omitted because of a lack of significance.

^b^Predictors having positive associations with the engagement in app or health outcomes.

^c^Predictors having negative associations with the engagement in app or health outcomes.

^d^N/A: not applicable.

Regarding the model performance of the three prediction models, the machine learning approaches successfully predicted the engagement rate (mean *R^2^* 0.416, SD 0.006), short-term weight change (mean *R^2^* 0.382, SD 0.015), and long-term weight change (mean *R^2^* 0.590, SD 0.011). In predicting long-term weight change, approximately 59% of the outcome variance was explained by the prediction model. In summary, these model performances suggest that the multivariate profiles in conventional and digital phenotypes provide phenotypes that are significantly associated with engagement and health outcomes.

## Discussion

### Principal Findings

Using a machine learning approach based on elastic net regression, we successfully demonstrated the applicability of the conceptual paradigm with complex dimensions of how in-app engagement is formed and affects health outcomes. This study showed that mobile apps’ engagement was significantly associated with health outcomes, even 4 months after the cessation of digital interventions. We also found that both conventional motivation (before the intervention) and in-app motivation (during the intervention) were closely related to both engagement and clinical outcomes. Multiple aspects of motivation before and during the intervention could be used to predict engagement and health outcomes. Furthermore, both engagement and health outcomes are associated with multivariate psychological indices patterns, such as behavioral, cognitive, emotional, and motivational components, driven by regularized multivariate profiles obtained using the machine learning approach. From the results, we conclude that individuals’ psychological states are the primary elements that influence engagement and health outcomes.

This study makes a clear implication on how engagement with apps influences clinical outcomes. Our finding that a higher frequency of logging into an app drives more significant improvements in health outcomes during the active intervention period is consistent with previous studies [27,28]. However, a notable finding in this study is that those who logged into the app more frequently also showed more favorable health outcomes after the cessation of the active intervention period. These results indicate that engagement is paramount to the app’s potential effectiveness for behavior change, leading to a change in symptomatology. Thus, it is feasible for clinicians and users to predict their health outcomes according to the intensity of their participation in apps.

Digital interventions via apps are not the only realm in which engagement is an issue. Both face-to-face and digital interfaces encounter difficulties in maintaining adherence and engagement with monitoring, medications, and psychotherapies [29]. Digital therapeutics are beneficial for monitoring and analyzing real-time data and reaching out to users without barriers in space and time; however, they are more applicable to offer immediate feedback and prevent attrition than face-to-face clinics. From this perspective, a previous meta-analysis claimed that integrating a human factor into the treatment is an actionable strategy to alleviate dropout rates in the digital intervention [30]. Our result is also supportive in that the number of messages (interaction frequency between the user and therapist) showed the highest positive standardized coefficient with engagement with the app. Taken together, we suggest that human feedback is involved in the development of digital therapeutics to strengthen the engagement rate, leading to greater clinical efficacy.

For the first time, this study evaluated the multiple dimensions of motivation at two different periods: before (conventional motivation) and during (in-app motivation) the intervention [31,32]. Previous studies assessed motivation at several time points but only one dimension (ie, usability or satisfaction with digital intervention) [33,34]. Furthermore, other studies measured multiple dimensions of motivation (ie, satisfaction, acceptability, and usability) but only assessed one period (ie, after the digital intervention) [35,36]. These previous designs have limitations in reflecting the users’ true motivation and predicting both engagement and clinical outcomes. According to our results, the common predictors of both engagement rate and health outcomes were in-app motivational phenotypes, referred to as satisfaction with the intervention, desire to improve health outcomes, and self-confidence. The level of self-esteem at baseline was also a common predictor of both engagement and health outcomes. Moreover, before implementing the intervention, the level of motivation was strongly related to health outcomes in both the short- and long-term courses. Altogether, these results suggest that motivation is the main component that determines engagement and health outcomes.

Previously, pragmatic qualities, systematic flow, satisfaction, usability, and esthetics were known as the major contributors to digital therapeutic engagement [7,27,29]. These previous results only serve as a basis for preliminary hypotheses on what may force engagement with apps. Few studies have examined engagement based on individuals’ interactions with various intervention elements such as frequency of access, an average of steps, article views, and message views [28,37,38]. However, it is still challenging to establish a standardized approach to assess the engagement of these phenotypes because of various factors, such as diverse technological aspects, different intervention exposure times, and individual characteristics. Thus, we suggest measuring the multiple aspects of motivation directly before and during the intervention to predict dropout and give each participant individualized attention.

This is the first study to categorize diverse digital phenotypes into four different constructs: behavior, cognition, emotion, and motivation. This allows a comprehensive understanding of the nature of behavior change, which is closely related to the engagement and clinical outcomes of digital interventions. We suggest that the behavioral phenotypes (calorie density of food, snack time of the day, amount of food intake per meal, and frequency of message interactions with the therapists), emotional phenotypes (irritated, bored, and depressed), and motivational phenotypes (satisfaction, will, and confidence) are the favorable phenotypes for predicting the engagement in app and health outcomes. However, none of the cognitive phenotypes were capable of engaging in the app. The phenotypes predicting the health outcomes were similar but not identical to the engagement because the amount of nutritional intake was included instead of the amount of food intake per meal for the behavioral phenotypes, and depressive moods were excluded from the emotional phenotypes. These findings imply that not only users’ physical participation in a specific target behavior (eg, logging food diary and number of steps) and behavior in digital spaces (eg, number of accesses) but also the user’s psychological conditions (eg, emotion and motivation) are relevant to engagement and clinical outcomes.

To the best of our knowledge, this is the first study to apply a machine learning approach to provide relevant insights into improving both the adherence and clinical outcomes of digital interventions. Although previous mHealth intervention studies have shown that user engagement is critical to clinical outcomes, little effort has been made to conceptualize and estimate it. The major reason is that only a few mHealth programs predominantly use the applicable data to investigate participants’ engagement or to examine its correlation with primary outcomes. However, we demonstrated the whole framework of how different types of phenotypes at baseline and during the intervention carry out in-app engagement and health outcomes. We used machine learning strategies with digital phenotypes to find an applicable model to predict intervention adherence for the first time.

This is also the first study to examine the determinants of significant weight changes from digital interventions. In addition, our first attempt to explore the phenotypes in two different periods (at baseline and during the intervention) and categorize them into four distinctive dimensions (behavior, cognition, emotion, and motivation) presents more comprehensive perceptions of engagement mechanisms and clinical outcomes. Finally, this study applied two specific methods, in-app and a web-based survey, for the first time to collect sufficient data, which led us to explore various components attaining favorable solutions for the issue of engagement and clinical efficacy in digital therapeutics. Using digital phenotypes and enhancing our insight into them to promote management will involve refined approaches for choosing and investigating diverse digital health data streams in a definite manner.

### Limitations

This study had several limitations. First, all participants received cognitive behavioral therapy, so it lacked a control group that did not receive any intervention. Second, the number of participants was relatively small, which might not be sufficient for a reliable interpretation. However, as we extracted multivariate profiles to predict engagement and health outcomes, we remedied the shortage by using a machine learning approach. Furthermore, as this study explores the challenging concept of digital interventions, a small number of participants are still tolerable to apply the machine learning analysis [39]. Third, considering the relatively small sample size, the leave-one-out cross-validation may be sensitive to outliers in the data set. Furthermore, our study is somewhat exploratory, limited by the small sample size, which requires further investigation with large data sets to consolidate the validity of our findings. Finally, the experiment did not track longitudinal changes in health outcomes in the app.

### Conclusions

Using a machine learning approach, we successfully established and validated an intuitive analytic strategy and provided visualization with a multiplex component paradigm of causality underlying digital psychotherapy on health outcomes. Our results revealed a key mechanism of psychological features interacting with multiple dimensions of motivation, which induce engagement in the app and enhance clinical efficacy. We expect that this study will play a significant role in establishing the most practical and effective mHealth intervention model, a vital insight for precision digital medicine.

## Appendix

Multimedia Appendix 1 Cross-validation results on the model performance of three elastic net models based on different mixing parameter values (α), the categorization of digital phenotypes and items used for each phenotype, and β estimates for conventional and digital phenotypes on the predictions of engagement and health outcomes.

Multimedia Appendix 2 CONSORT-EHEALTH (V 1.6.1) - Checklist.

## Abbreviations

BSQ: Body Shape Questionnaire
CDI: calorie density index
dCBT: digital cognitive behavioral therapy
mHealth: mobile health
NRF: National Research Foundation
RMSE: root mean squared error
SIMS: Situational Motivation Scale
