# Supplementary material for: Machine Learning Analysis to Identify Digital Behavioral Phenotypes for Engagement and Health Outcome Efficacy of an mHealth Intervention for Obesity: Randomized Controlled Trial
Source: J Med Internet Res. 2021 Jun 24;23(6):e27218. doi: 10.2196/27218 (PMC8277339; doi:10.2196/27218)
Supplement: Multimedia Appendix 1 [file jmir_v23i6e27218_app1.docx]

# Supplementary materials

**Figure S1.** Cross-validation results on the model performance of three elastic net models based on different mixing parameter values (alpha). Each point indicates the mean value of simulated root mean squared errors (RMSE) for each alpha value, and the error bar indicates 95% quantile ranges on the simulated RMSEs. The alpha value with the minimum RMSE was chosen for each model.


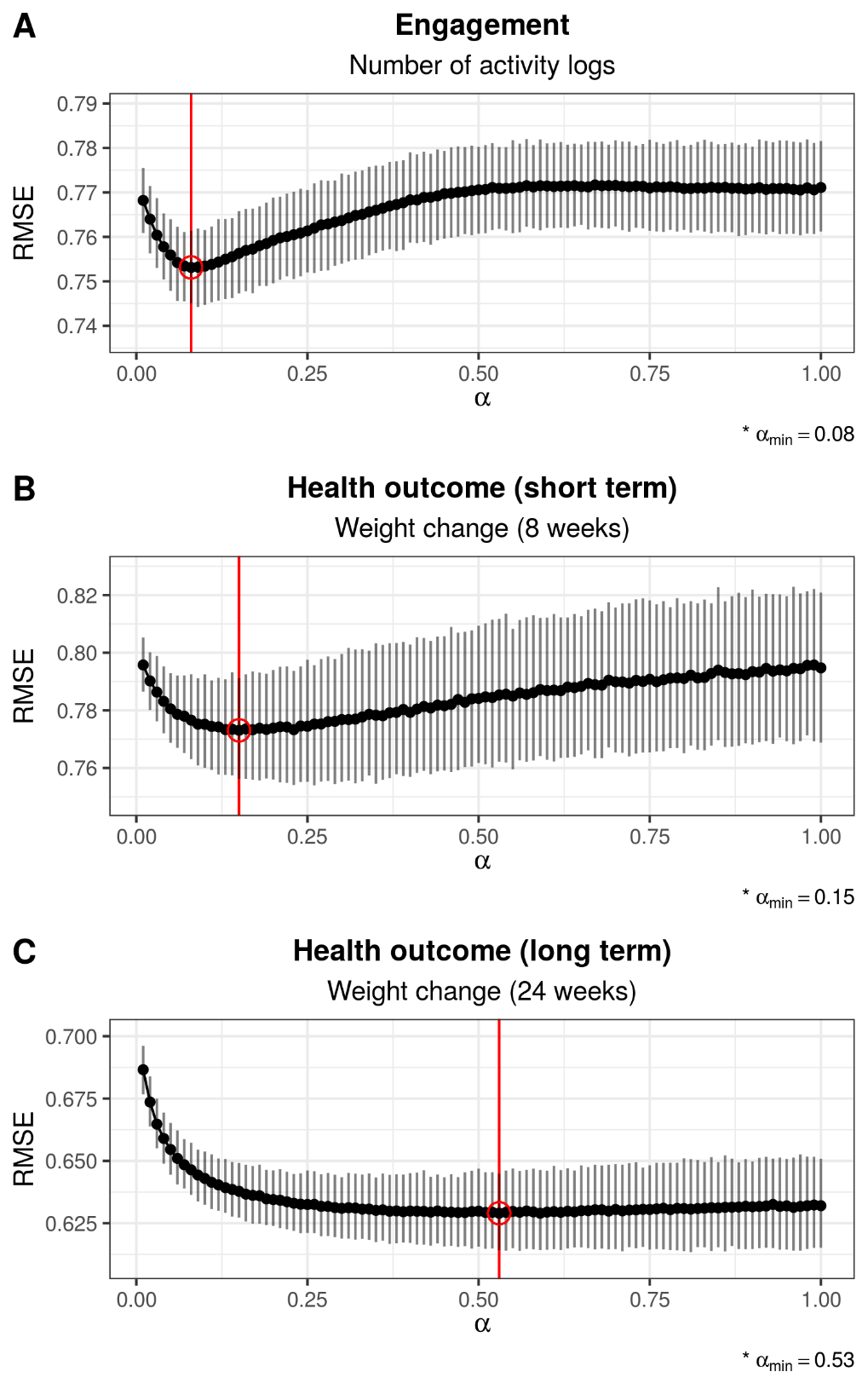


**Table S1.** The categorization of digital phenotypes and items used for each phenotype.

| **Part 1. Behavior** | | | |
| --- | --- | --- | --- |
| **The Place to Eat** | **Main Question** | **Sub-Categories** | **Responses** |
|  | Where did you eat? | Breakfast | 1 (Skipped the meal)  2 (At home)  3 (At the office)  4 (At the restaurant)  5 (In the traffic)  6 (Others) |
|  |  | Morning snack |  |
|  |  | Lunch |  |
|  |  | Afternoon snack |  |
|  |  | Dinner |  |
|  |  | Late-night snack |  |
| **The Time Period to Eat** | **Main Question** | **Sub-Categories** | **Responses** |
|  | What time of the day did you eat? | Breakfast | 1 (Skipped the meal)  2 (6:00am~)  3 (7:00am~)  4 (8:00am~)  5 (9:00am~)  6 (10:00am~)  7 (11:00am~)  …. 17 (21:00pm~),  18 (Others) |
|  |  | Morning snack |  |
|  |  | Lunch |  |
|  |  | Afternoon snack |  |
|  |  | Dinner |  |
|  |  | Late-night snack |  |
| **The Speed of Eating** | **Main Question** | **Sub-Categories** | **Responses** |
|  | How long did you take to eat? | Breakfast | 1 (Skipped the meal)  2 (less than 5mins)  3 (5mins. ~ 10mins.)  4 (10mins. ~ 15mins.)  5 (15mins. ~ 20mins.)  6 (more than 20mins.) |
|  |  | Morning snack |  |
|  |  | Lunch |  |
|  |  | Afternoon snack |  |
|  |  | Dinner |  |
|  |  | Late-night snack |  |
| **The Type of Food** | **Main Question** | **Sub-Categories** | **Responses** |
|  | What type of food did you eat? | Breakfast | 1 (Skipped the meal)  2 (Liquid)  3 (Fruits/Finger food)  4 (Full set diet) |
|  |  | Morning snack |  |
|  |  | Lunch |  |
|  |  | Afternoon snack |  |
|  |  | Dinner |  |
|  |  | Late-night snack |  |

| **Part 2. Cognition** | | |
| --- | --- | --- |
| **Main Question** | **The list of thoughts** | **Checked** |
| The thoughts that came to your mind during the day | - It is free, so you can eat a lot. - If you leave food, it is a waste, so you must eat it all.  - There is no difference between eating less or more since I have already exceeded the recommended calories per day.  - If I do not eat, he/she will be disappointed.  - If I become thinner, other people will like me more.  - If I leave food, other people will think I am spoiled.  - When I gain weight, others will ignore me.  - If I do not eat, other people will think I am timid.  - I cannot help it since I failed my diet again.  - I failed to lose weight since I could not do the exercise I was supposed to do today.  - I am ruined since I passed the recommended calories per day.  - I am going to gain a lot of weight again since I ate OOO.  - It is okay if I skip dinner.  - I can have a lot for dinner since I ate a little for lunch.  - It is okay to eat a lot since I am going to exercise.  - I can have as much as I want for lunch since I skipped my breakfast.  - Eating a lot of fruits is okay.  - If I enjoy eating, it’s 0 kcal.  - If you eat as much as you want, the stress can be resolved.  - Spicy food does not make you gain weight. | 0 – No  1 - Yes |

| **Part 3. Emotion** | | |
| --- | --- | --- |
| **Main Question** | **The list of emotions** | **Responses** |
| **How did you feel today?** | Irritated | VAS 0-100 |
|  | Lonely |  |
|  | Anxious |  |
|  | Bored |  |
|  | Depressed |  |

| **Part 4. Motivation** | | |
| --- | --- | --- |
| **Main Question** | **The list of dimensions in motivation** | **Responses** |
| How much weight do you wish to lose? | Will | VAS 1-10 |
| How important is it to lose body weight? | Rank of importance |  |
| How confident do you feel about losing weight? | Confidence |  |
| How helpful is this weight loss program to you? | Satisfaction |  |

**Table S2.** Beta estimates for conventional and digital phenotypes on the predictions of engagement and health outcomes. Mean and quantile values (2.5% and 97.5%) are emphasized if the intervals do not range over zero. Empty cells indicate trivial cases meaning that all three values equal to zero.

|  |  |  | **Engagement** | | | **Health outcome  (short-term)** | | | **Health outcome  (long-term)** | | |
| --- | --- | --- | --- | --- | --- | --- | --- | --- | --- | --- | --- |
|  |  |  | Number of activity logs | | | Weight loss (8 weeks) | | | Weight loss (24 weeks) | | |
| **Category** |  | **Phenotype** | **Mean** | **2.5%** | **97.5%** | **Mean** | **2.5%** | **97.5%** | **Mean** | **2.5%** | **97.5%** |
| **Conventional Phenotypes** | | AuthTh | 0.000 | -0.001 | 0.002 | 0.000 | -0.001 | 0.001 | 0.000 | -0.002 | 0.003 |
|  |  | Body Satisfaction | **-0.009** | **-0.012** | **-0.006** |  |  |  | **-0.012** | **-0.020** | **-0.005** |
|  |  | Emotional Eating | 0.000 | -0.001 | 0.001 | **-0.017** | **-0.027** | **-0.007** | 0.000 | -0.002 | 0.002 |
|  |  | Environmental Eating | **0.014** | **0.010** | **0.018** | **0.071** | **0.060** | **0.083** | 0.001 | -0.001 | 0.003 |
|  |  | Restrictive Eating |  |  |  | 0.000 | 0.000 | 0.001 | **0.099** | **0.082** | **0.115** |
|  |  | Depression | 0.001 | -0.002 | 0.003 | 0.000 | -0.001 | 0.001 | 0.001 | -0.005 | 0.007 |
|  |  | Self-esteem | **-0.055** | **-0.062** | **-0.049** | **-0.015** | **-0.022** | **-0.009** | **-0.173** | **-0.187** | **-0.159** |
|  |  | Motivation |  |  |  | **0.102** | **0.089** | **0.114** | **0.064** | **0.057** | **0.070** |
|  |  | Anxiety |  |  |  | **-0.009** | **-0.012** | **-0.006** | -0.003 | -0.008 | 0.001 |
|  |  | Food Addiction |  |  |  | 0.000 | -0.001 | 0.001 | **-0.040** | **-0.052** | **-0.029** |
| **Digital Phenotypes** | Behavioral | Breakfast | **0.002** | **0.002** | **0.003** | 0.000 | -0.001 | 0.000 | 0.000 | -0.001 | 0.001 |
|  |  | Morning Snack | **0.024** | **0.020** | **0.029** |  |  |  | 0.000 | -0.001 | 0.001 |
|  |  | Lunch | **-0.015** | **-0.018** | **-0.012** | **-0.002** | **-0.004** | **0.000** | **-0.008** | **-0.014** | **-0.002** |
|  |  | Afternoon Snack |  |  |  | **0.014** | **0.004** | **0.025** | 0.000 | -0.001 | 0.001 |
|  |  | Dinner | **-0.002** | **-0.002** | **-0.002** | **-0.004** | **-0.006** | **-0.003** | 0.001 | -0.005 | 0.007 |
|  |  | Evening Snack | **-0.034** | **-0.038** | **-0.030** | 0.000 | -0.001 | 0.000 | **-0.112** | **-0.118** | **-0.106** |
|  |  | Carb | 0.000 | -0.001 | 0.001 | **-0.081** | **-0.093** | **-0.069** | **-0.252** | **-0.275** | **-0.230** |
|  |  | Protein | 0.000 | -0.001 | 0.001 |  |  |  | 0.000 | -0.001 | 0.001 |
|  |  | Fat | 0.000 | -0.001 | 0.002 | **-0.005** | **-0.007** | **-0.004** | **-0.010** | **-0.014** | **-0.005** |
|  |  | Sugar | **0.014** | **0.011** | **0.017** | 0.001 | -0.001 | 0.002 | 0.000 | -0.002 | 0.002 |
|  |  | Sodium | 0.000 | -0.001 | 0.000 | **-0.023** | **-0.026** | **-0.020** | 0.001 | -0.005 | 0.007 |
|  |  | Low Cal Food | **0.065** | **0.062** | **0.069** | **0.045** | **0.041** | **0.050** | 0.000 | -0.002 | 0.002 |
|  |  | Moderate Cal Food | **0.059** | **0.052** | **0.065** |  |  |  | 0.000 | 0.000 | 0.001 |
|  |  | High Cal Food | **-0.115** | **-0.119** | **-0.110** | **-0.103** | **-0.116** | **-0.091** | -0.001 | -0.003 | 0.001 |
|  |  | Exercise |  |  |  |  |  |  | -0.001 | -0.007 | 0.004 |
|  |  | Steps | 0.000 | -0.001 | 0.001 | 0.000 | -0.001 | 0.000 | **-0.002** | **-0.003** | **-0.001** |
|  |  | InteractionFreq | **0.080** | **0.074** | **0.086** | **0.043** | **0.036** | **0.050** | 0.000 | -0.001 | 0.002 |
|  | Cognitive | BW-AuthTh | 0.000 | -0.001 | 0.001 |  |  |  | 0.002 | -0.002 | 0.005 |
|  | Emotional | Bored | **0.003** | **0.002** | **0.004** | **0.005** | **0.004** | **0.006** | 0.000 | -0.001 | 0.002 |
|  |  | Depressed | **0.016** | **0.015** | **0.017** | 0.000 | 0.000 | 0.001 | 0.000 | -0.001 | 0.001 |
|  |  | Irritated | **0.001** | **0.001** | **0.002** | **0.005** | **0.004** | **0.006** |  |  |  |
|  |  | Lonely |  |  |  |  |  |  |  |  |  |
|  |  | Nervous |  |  |  |  |  |  | -0.002 | -0.012 | 0.009 |
|  | Motivational | Confidence | **0.072** | **0.070** | **0.074** | **0.068** | **0.061** | **0.075** | **0.132** | **0.126** | **0.138** |
|  |  | Importance |  |  |  | 0.000 | -0.001 | 0.001 | 0.000 | -0.001 | 0.001 |
|  |  | Satisfaction | **0.052** | **0.051** | **0.053** | **0.047** | **0.041** | **0.053** | **0.078** | **0.069** | **0.087** |
|  |  | Will | **0.053** | **0.051** | **0.054** | **0.024** | **0.021** | **0.026** | **0.110** | **0.100** | **0.120** |
